# Modelling the effects of global climate change on Chikungunya transmission in the 21^st^ century

**DOI:** 10.1038/s41598-017-03566-3

**Published:** 2017-06-19

**Authors:** Nils B. Tjaden, Jonathan E. Suk, Dominik Fischer, Stephanie M. Thomas, Carl Beierkuhnlein, Jan C. Semenza

**Affiliations:** 1 Department of Biogeography, University of Bayreuth, Bayreuth, Germany; 2 European Centre for Disease Prevention and Control (ECDC), Stockholm, Sweden; 3 Technical University of Munich (TUM), Munich, Germany

## Abstract

The arrival and rapid spread of the mosquito-borne viral disease Chikungunya across the Americas is one of the most significant public health developments of recent years, preceding and mirroring the subsequent spread of Zika. Globalization in trade and travel can lead to the importation of these viruses, but climatic conditions strongly affect the efficiency of transmission in local settings. In order to direct preparedness for future outbreaks, it is necessary to anticipate global regions that could become suitable for Chikungunya transmission. Here, we present global correlative niche models for autochthonous Chikungunya transmission. These models were used as the basis for projections under the representative concentration pathway (RCP) 4.5 and 8.5 climate change scenarios. In a further step, hazard maps, which account for population densities, were produced. The baseline models successfully delineate current areas of active Chikungunya transmission. Projections under the RCP 4.5 and 8.5 scenarios suggest the likelihood of expansion of transmission-suitable areas in many parts of the world, including China, sub-Saharan Africa, South America, the United States and continental Europe. The models presented here can be used to inform public health preparedness planning in a highly interconnected world.

## Introduction

Chikungunya is a mosquito-borne arboviral disease transmitted by *Aedes* species mosquitoes, notably *Aedes aegypti* and *Aedes albopictus*. Historically endemic in tropical climates such as in Africa, Southeast Asia and the Indian subcontinent, events of the past decade have led to a substantial geographic expansion of the disease. In 2005-06, an outbreak with nearly 1.4 million reported cases occurred in India^1^, and another large outbreak on La Réunion led to over 250,000 reported cases^2^. Thereafter, autochthonous transmission by *Ae. albopictus* was recorded in temperate continental Europe for the first time in northern Italy in 2007^3^, followed by southern France in 2010^4^ and 2014^5^. Chikungunya transmission has recently also occurred in China^6^, Papua New Guinea and New Caledonia^7^. In December 2013, Chikungunya arrived in the Americas on the Caribbean island of St. Martin^8, 9^, from which it subsequently spread to at least 45 countries and territories, leading to at least 1.7 million suspected cases. This illustrates how the disease continues to disperse internationally and pose a threat to public health.

Numerous factors played a role in the global spread of Chikungunya. Adaptive mutations in the Chikungunya genome enabled the East/Central/South African (ECSA) strain to be more easily transmitted by *Ae. albopictus*, contributing to the outbreak in La Réunion^10^ and, subsequently, to outbreaks in south Asia and Italy. Globalization in trade and travel, meanwhile, have facilitated the geographic expansion of *Ae. albopictus* ^11^ and have increased the possibility that travellers infected with Chikungunya could come into contact with competent *Aedes* mosquito vectors^12, 13^.

Although global interconnectivity ensures a continued risk for importations of Chikungunya into regions with competent mosquito vectors, until very recently there were no global distribution models for this viral disease, and comparatively little research identifies global regions of climatic suitability for Chikungunya transmission. It is, however, well known that climate affects growth, survival and abundance of the two primary vectors for Chikungunya, *Ae. aegypti* and *Ae. albopictus* ^14^. Both field and laboratory experiments demonstrate that survival of both of these mosquito species is affected by lower and upper temperature thresholds^15^. Precipitation is another important factor influencing the availability of microhabitats for oviposition and larval development: heavy rainfalls – which are increasing in frequency due to climate change in some areas – have increased the abundance of *Ae. albopictus*, thereby increasing the risk of Chikungunya transmission in southern France in 2014^5^. Projections of the global distribution of *Ae. albopictus* and *Ae. aegypti* under climate change scenarios generally anticipate expansions in eastern North America, Central Africa, northern and eastern Australia, and East Asia^14^. Regional European models of *Ae. albopictus* under climate change scenarios suggest that climatic suitability will generally increase and populations expand northwards in the upcoming decades^16–18^.

While several epidemiological models exist for Chikungunya, global climate change models for the disease so far solely focus on vector distribution (with one exception^19^). One limiting factor is the knowledge gap about the effect of temperature on the extrinsic incubation period (EIP) of Chikungunya in both *Ae. albopictus* and *Ae. aegypti*. Present-day models for Chikungunya in Europe and the United States overcame this challenge through approximations based on field data^19^ or drawing parallels to similar diseases such as dengue^20^. One alternative approach that obviates the need to model the complex interactions of extrinsic and intrinsic factors related to Chikungunya transmission is correlative niche modelling, which treats the disease as a species with a specific environmental niche. This includes environmental effects on the pathogen (such as ambient temperature affecting the virus’ replication rate in the ectothermic vector’s body) as well as vector distribution. Commonly applied for species distribution models of disease vectors^18^ as well as in conservation biology, this approach has successfully been applied to dengue^21^, Chikungunya^22^, Zika^23^ and other diseases^24–26^.

In this study, geospatially reported cases of Chikungunya were related to climatic factors so as to deduce the most influential climatic variables governing Chikungunya transmission. The characteristics of this niche were then used to assess the current global suitability for Chikungunya. Thereafter, the RCP 4.5 and RCP 8.5^27^ climate change scenarios were used to project how the global suitability for Chikungunya transmission might change in the future. In this context, high “climatic suitability” indicates an increased potential for Chikungunya transmission to occur but does not necessarily mean that actual outbreaks will take place, as public health control measures and overall levels of socioeconomic development could serve as mitigating measures.

**Figure 1:**
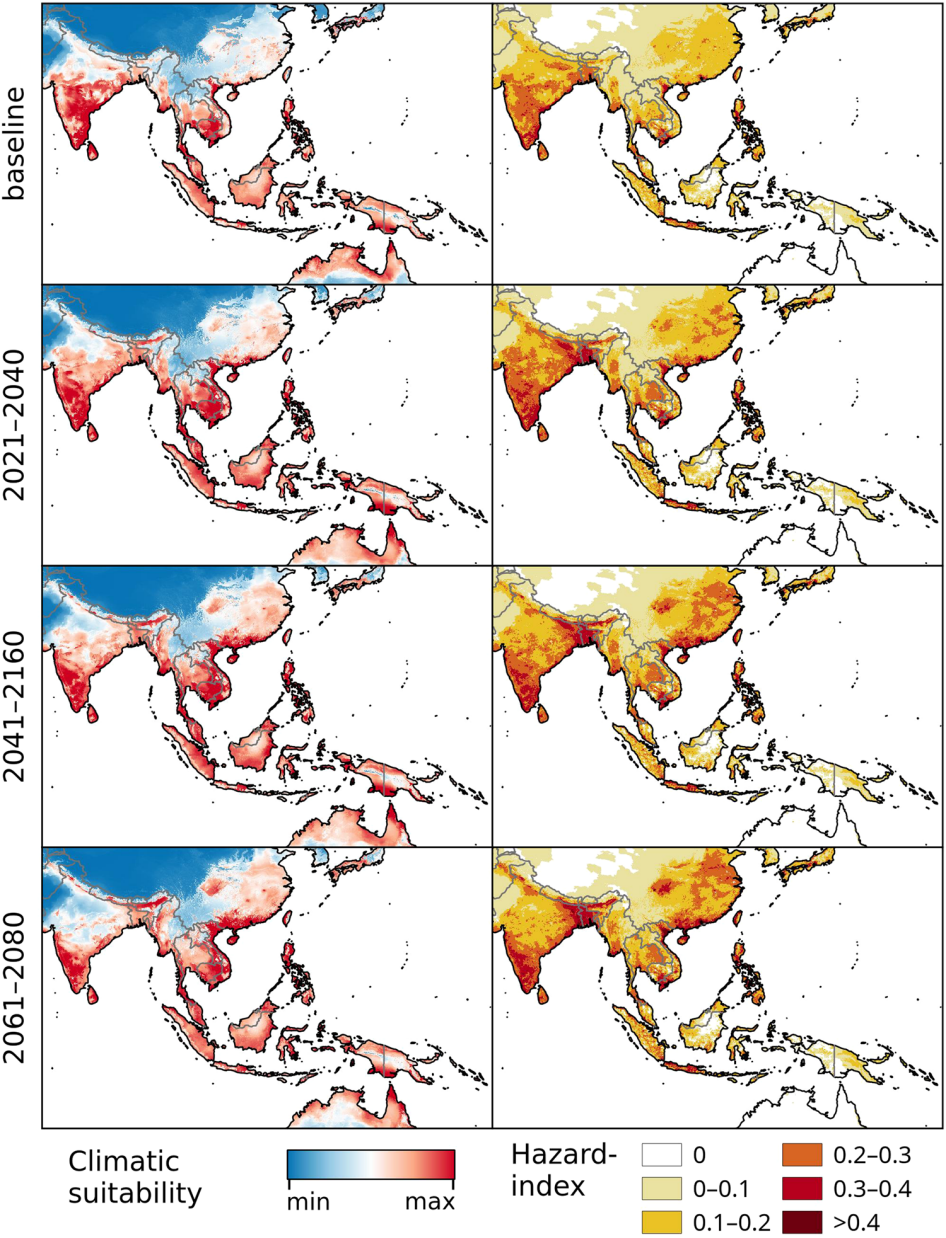
Chikungunya under the baseline and RCP 8.5 climate change scenarios in Asia and Australasia. Left: Climatic suitability, right: hazard index. Climate change scenarios represent the mean model output obtained through the 5 GCMs. Climatic suitability output is scaled to the over-all global minimum (0) and maximum (0.623) values observed in any model. Maps were generated using the “raster” package in R 3.3.2 (https://www.r-project.org/) and QGIS 2.8.1 (https://www.qgis.org/).

**Figure 2:**
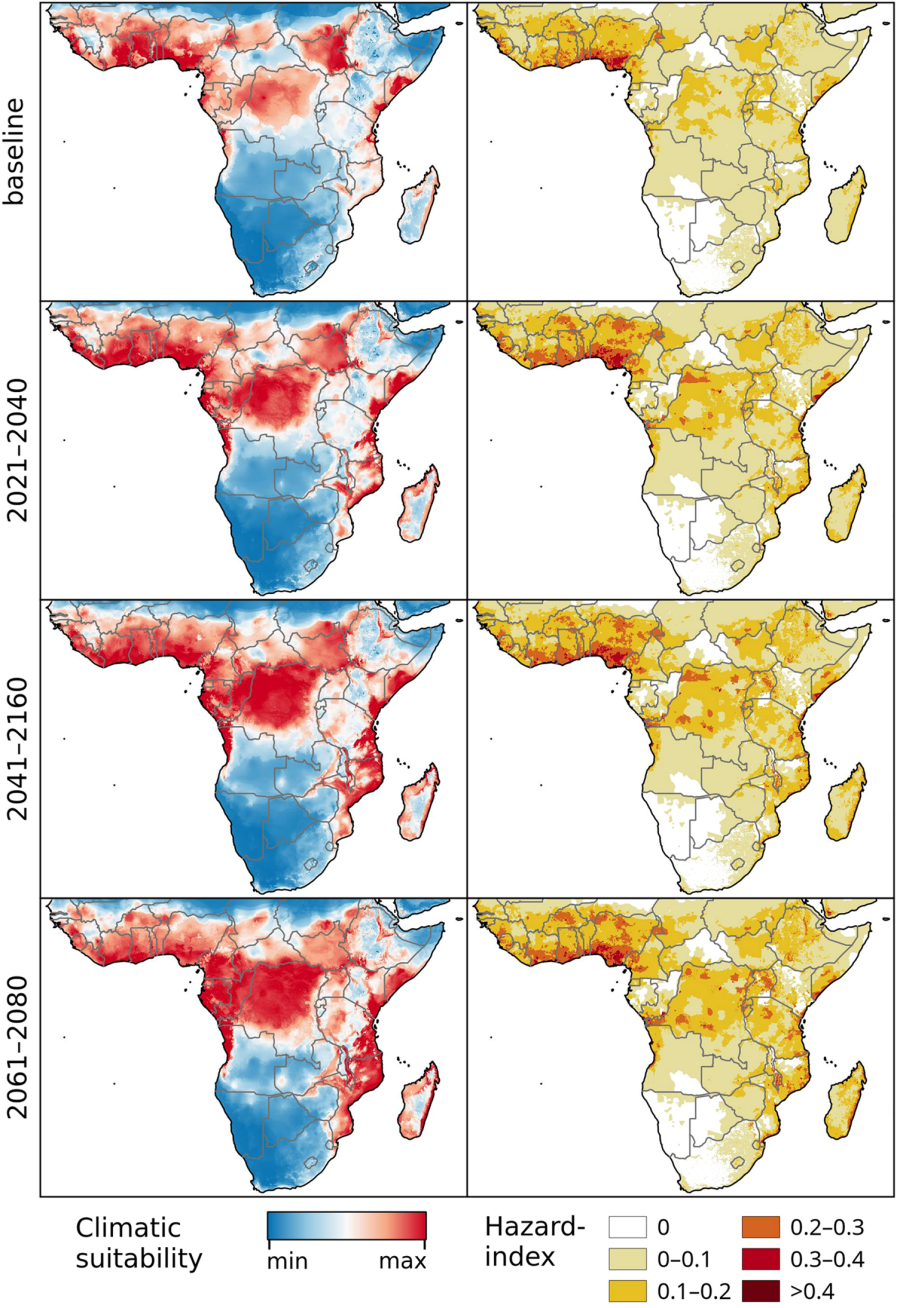
Chikungunya under the baseline and RCP 8.5 climate change scenarios in Africa. Left: Climatic suitability, right: hazard index. Climate change scenarios represent the mean model output obtained through the 5 GCMs. Climatic suitability output is scaled to the over-all global minimum (0) and maximum (0.623) values observed in any model. Maps were generated using the “raster” package in R 3.3.2 (https://www.r-project.org/) and QGIS 2.8.1 (https://www.qgis.org/).

**Figure 3:**
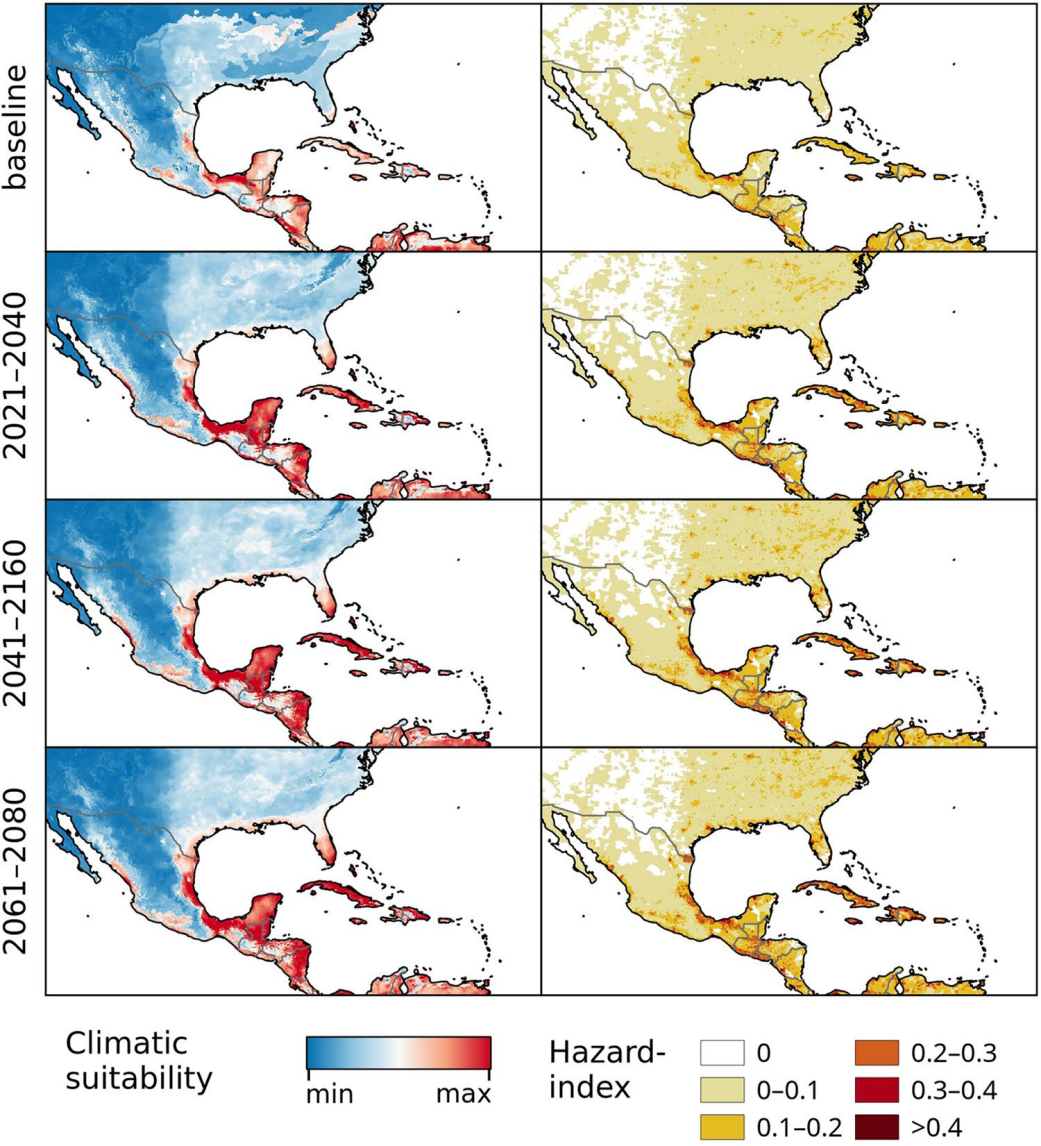
Chikungunya under the baseline and RCP 8.5 climate change scenarios in North- and Central America. Left: Climatic suitability, right: hazard index. Climate change scenarios represent the mean model output obtained through the 5 GCMs. Climatic suitability output is scaled to the over-all global minimum (0) and maximum (0.623) values observed in any model. Maps were generated using the “raster” package in R 3.3.2 (https://www.r-project.org/) and QGIS 2.8.1 (https://www.qgis.org/).

**Figure 4:**
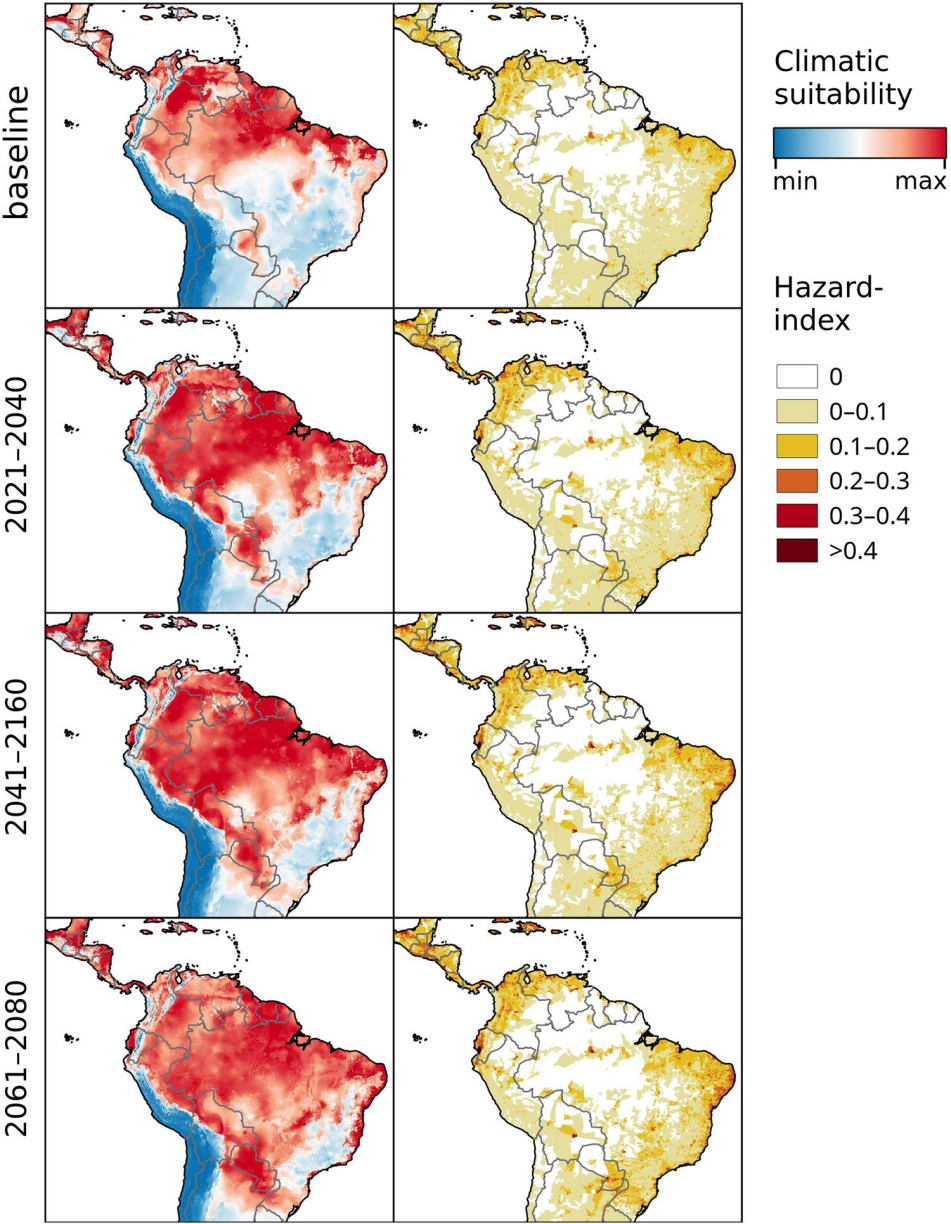
Chikungunya under the baseline and RCP 8.5 climate change scenarios in South America. Left: Climatic suitability, right: hazard index. Climate change scenarios represent the mean model output obtained through the 5 GCMs. Climatic suitability output is scaled to the over-all global minimum (0) and maximum (0.623) values observed in any model. Maps were generated using the “raster” package in R 3.3.2 (https://www.r-project.org/) and QGIS 2.8.1 (https://www.qgis.org/).

**Figure 5:**
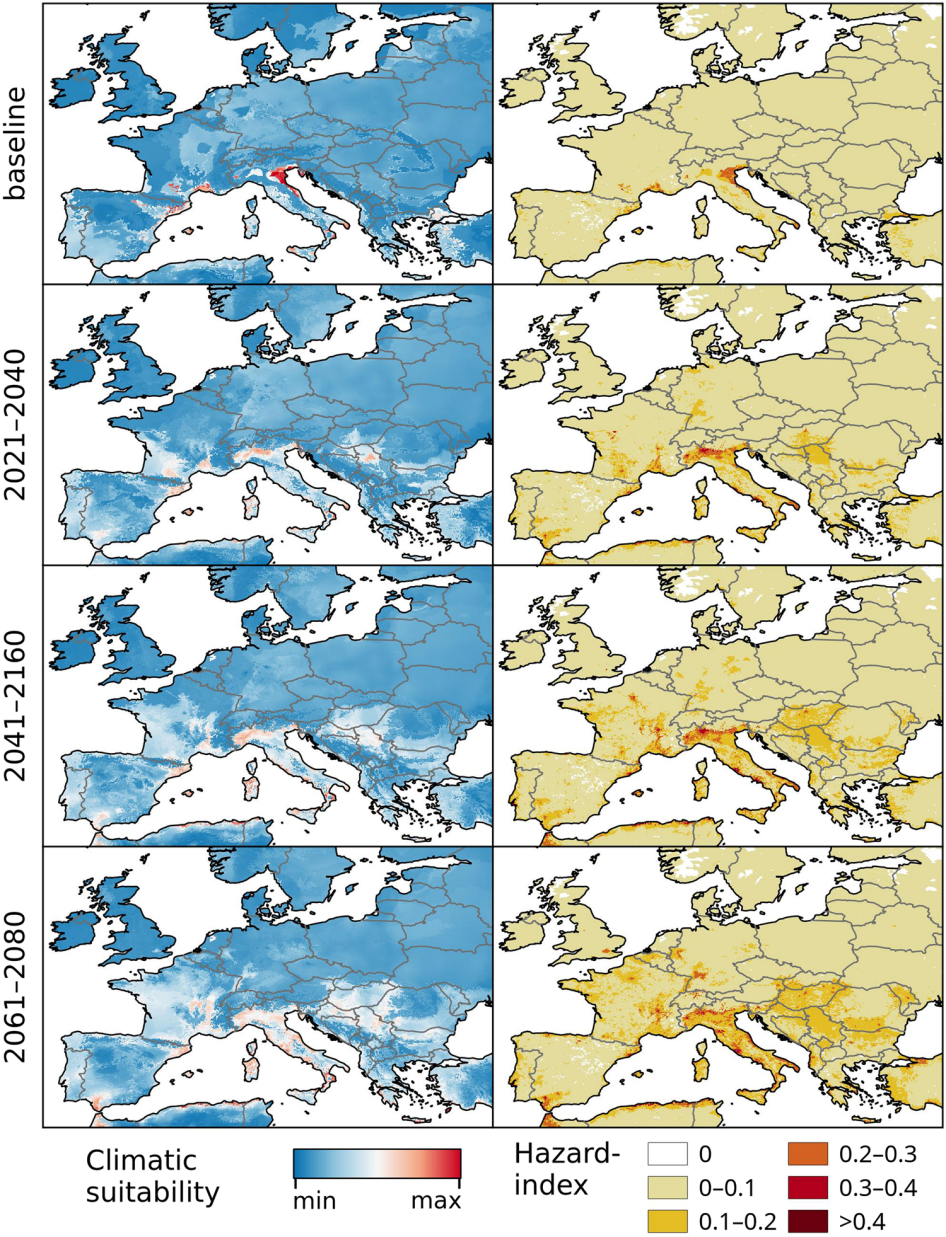
Chikungunya under the baseline and RCP 8.5 climate change scenarios in Europe. Left: Climatic suitability, right: hazard index. Climate change scenarios represent the mean model output obtained through the 5 GCMs. Climatic suitability output is scaled to the over-all global minimum (0) and maximum (0.623) values observed in any model. Maps were generated using the “raster” package in R 3.3.2 (https://www.r-project.org/) and QGIS 2.8.1 (https://www.qgis.org/).

The models developed in this study focus solely on the climatic suitability of Chikungunya transmission based upon five explanatory variables identified during the modelling process: Annual mean temperature, minimum temperature of the coldest month, mean temperature of the wettest quarter, mean temperature of the warmest quarter and annual precipitation. Present-day (or baseline) models for climatic suitability for Chikungunya transmission were developed (top-left panel, Figs 1–5) based on climate data from worldclim.com^28^.

Under the RCP 4.5 and RCP 8.5 climate change scenarios, climate suitability maps for Chikungunya transmission were developed for the time periods of 2021–2040, 2041–2060, and 2061–2080 (Figs 1–5 and S1–S5, left panels) based on data from 5 different global climate models. In addition, maps of Chikungunya hazard, which additionally account for human population densities, were developed (RCP 8.5 depicted in Figs 1–5, right panels).

## Results

Our models reflect the current global distribution of Chikungunya (Fig. S8), but also identify areas suitable for transmission that have not suffered from larger outbreaks in the past. These include regions in northern and southern Italy, southwest France, northeast Spain, large areas of sub-Saharan Africa, northern Australia and the southernmost tip of Florida in the United States.

Projections for two contrasting climate change scenarios (RCP 4.5, RCP 8.5) show rather similar global patterns in the suitability- and hazard maps that were generated in this study. However, the modelling results for the high emission scenario, RCP 8.5 indicate areas of higher climatic suitability and larger expanse of suitable areas. Nevertheless, we also find areas with declining suitability as well as spatial contraction of suitable areas.  In Asia, the models suggest that both climatic suitability and Chikgununya hazard will generally increase in large parts of China, which had been largely free of autochthonous Chikungunya transmission until the 2010 outbreak in the Guangdong Province^6^ (Figs 1, S1). India shows a gradual decrease in climatic suitability in its central regions, with persistently strong suitability continuing in the southern regions. Southeast Asia and northern Australia demonstrate strong transmission suitability throughout all time periods, with considerably lower hazard in much of Australia due to low population densities.

In Sub-Saharan Africa, climatically suitable areas are projected to increase within the 2021–2040 timeframe and remain relatively stable thereafter under both climate change scenarios (Figs 2, S2). Highly suitable regions include the Atlantic coast from Senegal through to mid Angola, and a belt beginning in West Africa and continuing through to South Sudan. Most of the Indian Ocean coastline is also projected to be suitable for Chikungunya, with the exception of the Horn of Africa and South Africa. The risk of autochthonous transmission will be principally restricted to the more populated coastal areas of Somalia, Tanzania, and Mozambique (Fig. 2, right panels).

The climatic suitability for Chikungunya transmission is projected to steadily increase in the Gulf Coast, southern Florida, Cuba, the Yucatan peninsula, Sinaloa, and across much of Central America under both higher and lower emission scenarios (Figs 3, S3). In South America, our models identify a southerly expansion of climatic suitability for Chikungunya transmission, with a marked increase in eastern Peru, eastern Bolivia, Paraguay, and much of central Brazil (Figs 4, S4). The high elevation areas of Chile, Bolivia, and Peru will remain unsuitable for Chikungunya transmission. Under the RCP 8.5 scenario, the over-all level of climatic suitability in South America is projected to decrease by the end of the century, when the climatic conditions will be too extreme for the vector species in many regions.

In Europe, both scenarios show a moderate expansion of climatic suitability across much of central Europe, notably in France and Italy (Figs 5, S5). Large areas surrounding the Rhine and Rhone rivers in Germany and France, respectively, are also projected to increase in suitability. However, some parts of the region of highest current suitability in northern Italy near the Adriatic coast are projected to experience a decline in suitability in both scenarios due to increased probabilities of summer droughts, which will reduce the habitat suitability for the vectors.

## Discussion

Neither climate change nor global interconnectivity show signs of abating^12, 29^. As such, Chikungunya is likely to remain an important public health preparedness priority in regions where it has already been introduced as well as in regions at the fringes of its current distribution.

This is, to our knowledge, the first global study on spatio-temporal patterns of potential Chikungunya transmission using the RCP 4.5 and RCP 8.5 climate change scenarios. The modelling algorithms applied in this study to generate spatially explicit hazard maps for established climate change scenarios and time steps are based upon a correlative niche modelling approach to identify global regions that may be climatically favourable for Chikungunya transmission.

There are, broadly speaking, two key approaches for modelling vector-borne diseases. One is mechanistic modelling, which requires a detailed parameterization of numerous intricate biological processes, such as mosquito breeding and survival rates, mosquito biting rates, and the extrinsic incubation period. Although these models are thorough and based upon clear biological processes, there are important limitations to this approach. One relates to the unavailability of empirical data for the parameterization of biological processes, which may be a particular challenge for diseases such as Chikungunya which are relatively understudied. Another limitation is that modelling biological processes alone may tend to lead to overestimations (i.e. false positives) of the impacts of climate change, because they do not account for socioeconomic contexts or potential public health control measures^30–32^. In contrast, correlative modelling approaches such as the one presented here have an advantage in situations in which biological processes are incompletely parameterized^33^, as the method obviates the need to model the many unknown parameters that affect the interactions between Chikungunya virus, its mosquito vectors and humans. The focus instead is *a priori* on the climatic characteristics that are common to global regions that have recorded Chikungunya transmission.

Nonetheless, as with all modelling approaches, there are limitations to correlative niche modelling as well. First, vector-borne disease transmission is very complex, involving drivers across a wide range of socioeconomic and climatic variables. In the models presented here, socioeconomic vulnerabilities and related driving forces of Chikungunya transmission are intentionally excluded because the whole array and the diversity of processes in different continents and countries cannot be feasibly modelled. The models may nonetheless be indirectly affected by socio-economic and public health factors which may either protect against or exacerbate Chikungunya transmission. For example, there is historic evidence for Chikungunya occurring in relatively cool sub-tropical climates (such as Charleston, South Carolina, USA)^34^, but due to vector control and other measures current cases in those regions are sparse. Similarly, our models do not attempt to consider future adaptive measures that might be undertaken to mitigate the risk of Chikungunya transmission. Instead, we present models that identify hazard through the combination of climatic suitability and population density (right panels, Figs 1–5 and S1–S5).

A second limitation relates to the climatic input data. While the climate data used for the baseline model and future projections represent the same climatic parameters (such as “minimum temperature of the coldest month”), the underlying input data and methods are different. The Worldclim dataset for the baseline model is interpolated from data measured by weather stations^28^, whereas the data used for future projections comes from global climate models (GCM) that simulate physical processes in the atmosphere numerically. Although the approach of using those two data sources together has been widely applied, the comparability between baseline and future models is restricted nevertheless.

Finally, although calculating values for the mean climatic suitability from the climatic projections obtained from 5 different GCMs generally helps to increase confidence in the globally detected patterns (see Fig. S6 for standard deviations), small-scale differences in projected climate may lead to local under-estimations of climatic suitability (Fig. S7). Global models are only capable of displaying large-scale patterns and are best used for identifying areas of concern which could be further examined by subsequent smaller-scale models that would be better capable of representing locally relevant factors, such as the abundance of mosquito breeding sites, efforts in vector control, and local public health surveillance, preparedness, and response measures related to Chikungunya.

In comparing our baseline models with other recently-published works on Chikungunya^22, 35^ and its vectors^36^, there are general agreements at large scales, albeit with smaller-scale differences. In Oceania, for example, our model (Fig. 1), the Chikungunya model by Nsoesie *et al*.^22^ as well as the vector models by Kraemer *et al*.^36^ all cover the same general suitability areas between India, southern Japan and northern Australia. However, the model by Nsoesie *et al*.^22^ predicts comparably low environmental suitability in India (from where large numbers of Chikungunya cases have been reported, compare Fig. S8 and supplementary data), south-eastern China, southern Japan and northern Australia. When compared to the models by Kraemer *et al*.^36^, our model corresponds more closely to the *Ae. aegypti* model than the *Ae. albopictus* model for this region, but with lesser projected climatic potential for Chikungunya in the northern parts of India, where Chikungunya cases are currently less common (Fig. S8).

In Sub-Saharan Africa, all of these models predict high suitability in the area between roughly Senegal, the Ethiopian Plateau, the Congo Basin and the mouth of the Congo River, as well as Madagascar and a strip along the eastern coast between Kenya and Swaziland. Suitable areas also include parts of Angola and Zambia in the two vector models by Kraemer *et al*.^36^, while our model (Fig. 2) and the *Ae. aegypti* model^36^ predict higher suitability closer to the Sahara Desert in the north.

In Central America, all models agree on the Caribbean Islands as well as the coastal regions of the mainland being suitable for Chikungunya transmission. With the exception of the models by Mordecai *et al*.^35^, all models agree on Chikungunya or its vectors, respectively, being largely absent from the Savannahs and Steppes of inland-Mexico.

In North America, our model predicts relatively low over-all climatic potential for Chikungunya transmission. However, the areas of relatively higher suitability closely match the combined patterns of *Ae. aegypti* and *Ae. albopictus* distribution in the United States, as represented by the models by Kraemer *et al*.^36^ While the model by Nsoesie *et al*.^22^ appears to predict the US to be less suitable than all other models, those produced by Mordecai *et al*.^35^ predict 3 weeks of potential transmission areas as far north as Edmonton (Canada). The latter is probably due to the omission of low-temperature effects on mosquito survival as a modelling parameter, as even short periods of hard frost can significantly increase mortality of diapausing and non-diapausing *Aedes* eggs^37^.

In South America, all models covering the region predict a wide-spread potential for Chikungunya and its vectors respectively. Complete absence of Chikungunya is predicted for the Andes, Atacama Desert and Patagonia by all models. The mechanistic models of Mordecai *et al*.^35^ deviate from all other models by suggesting up to 5 consecutive months of potential transmission in the dry desert climates south of Trelew, Argentina as well as in a narrow strip along the western coast as far south as Los Ángeles, Chile. This is most likely due to the omission of precipitation and low-temperature limits as explanatory variables, as the very dry climate reduces availability of breeding sites for the vectors. In all other regions, Chikungunya transmission is possible in all models, though the distribution of relatively high and low suitability differs vastly among models.

In Europe, our baseline model (Fig. 5) appears to predict the locations of the recorded outbreaks in Italy and France much more accurately than the model by Nsoesie *et al*.^22^ When compared with the *Ae. albopictus* model from Kraemer *et al*.^36^, areas of very high climatic potential for Chikungunya transmission are more locally constrained in our model. Their vector model identifies suitable climatic conditions in Portugal and south-western Spain as well as nearly all coastal regions along the Mediterranean Sea. While many of these regions are not identified as highly climatically suitable areas for Chikungunya transmission in our model, it must be noted that they still represent a raised potential for Chikungunya transmission and should not be interpreted as low-risk areas.

To summarise, the two niche-type models based on Chikungunya occurrences, namely ours and the one by Nsoesie *et al*.^22^, anticipate less Chikungunya transmission in temperate regions than the other ones. This may simply be a surveillance artefact: current records of Chikungunya transmission in these areas are comparably sparse, possibly because Chikungunya is not generally expected in these regions by public health practitioners, which would mean that there is a gap in surveillance and, consequently, that our models under-estimate Chikungunya hazard in these areas. Conversely, perhaps more plausibly, it could mean that there may be additional effects of temperature that prevent Chikungunya transmission but not vector presence. It is important to note that while it is generally assumed that the Extrinsic Incubation Period (EIP) for Chikungunya is shorter than for Dengue, there are to our knowledge no systematic laboratory or field studies on how the EIP for Chikungunya changes at moderate to low temperatures. Even for Dengue, which is relatively well-studied, data on this is sparse and partially problematic^38^.

The novel models presented here demonstrate projected shifts in the climatic suitability for Chikungunya globally over the next century to identify regions with comparatively high hazards of Chikungunya transmission. The models project a net global increase in climate suitability for Chikungunya transmission by 2100, albeit with some important exceptions. Given the continued expectation for rapid global viral spread of Chikungunya alongside significant projected climatic changes over the next century, the models presented here can substantially contribute to integrated planning processes linking climate change adaptation with public health preparedness for mosquito-borne diseases.

## Methods

We compiled a global database of ca. 700 geo-referenced localities of confirmed autochthonous Chikungunya virus transmission from Promedmail, literature records, PAHO- and CARPHA-reports as well as global and local news outlets up until January 2015 (Fig. S8 and supplementary data). The majority of these records (73%) came from Asia, followed by the Americas (16%), Africa (9%) and Europe (2%). For some countries, we were forced to use centroids (geographical centres) of districts as geo-located regions (e.g. Bhutan, India, Thailand and Reunion Island). This may either be due to the reporting system (in case that no detailed coordinates or cities were mentioned), or to major outbreaks affecting whole districts. After removing duplicates as well as locations with insufficiently precise coordinates or missing climatic data coverage, 615 localities remained for use in the modelling process.

Once presence records had been prepared, bioclimatic variables obtained from the “Bioclim” dataset for current climatic conditions of Worldclim^28^ at a spatial resolution of 5 arcmin. Bioclimatic variables are derived from monthly temperature and rainfall values in order to generate biologically meaningful variables, representing annual trends, seasonality and extreme or limiting environmental factors. Those bioclimatic variables were referred to those sites with presence records, using the Maximum Entropy algorithm implemented in Maxent 3.3.3k^39^. Maxent is a commonly used method for predicting species distributions based on environmental variables and capable of accounting for interactions between variables. Instead of absence data, Maxent uses so-called background samples randomly drawn from the environment surrounding the presence records, accounting for the possibility of incorporating data from locations where the modelled species occurs but was not recorded. The maximum distance to the occurrence records from within which these background samples are drawn must be carefully chosen in order to avoid over- and underfitting^40^. Methods for doing this based on biological criteria exist^41^, but are primarily geared towards single species of higher organisms and do not necessarily translate well for complex virus-vector-host systems. As the dispersal potential of both the pathogen and its vectors is large due to human traffic, we opted for a buffer-based approach for estimating this potential. We produced a series of test models using buffer zones with radii between 0.1 and 10°. The resulting maps were carefully examined for artefacts such as high climatic suitability being predicted for obviously unsuitable areas or being limited only to the immediate surroundings of presence records. In our case, a buffer zone with a radius of 3° gave the best results. In order to come up with the challenge of spatial autocorrelation in data and to avoid spatial clustering in those regions with high numbers of documented cases (quantity effect) we created a spatial bias file as outlined by Elith *et al*.^42^

Selection of bioclimatic variables to be used in the final model was done using the “Jackknife” utility implemented in Maxent^43^ on a test run with all 19 Bioclim variables offered by Worldclim. This measures the effect each input variable has on the model’s training gain when a) the variable is considered in isolation and b) in combination with other variables, when this specific variable is dropped from the subset. For highly covarying variables only the one showing most influential potential in the Jackknife was considered for the final model. Based on this, the 5 most influential variables were:

- Annual mean temperature (bio 1)
- Minimum temperature of the coldest month (bio 6)
- Mean temperature of the wettest quarter (bio 8)
- Mean temperature of the warmest quarter (bio 10)
- Annual precipitation (bio 12)

The final baseline model was fit with these variables, using Maxent at default settings with a maximum of 1000 iterations. A 10-fold cross-validation was conducted for model validation, consisting of 10 separate runs with different sets of training data (used for fitting the model) and test data (used for testing model performance). Models were evaluated using partial receiver operating characteristics^44^, using 1000 bootstrapping iterations on 50% of the test data using an expected error rate of 5%. AUC ratios consistently were significantly larger than 1, suggesting good model performance.

In the following step, the baseline model was used for future projections under the IPCC-5 RCP 4.5 and 8.5 climate change scenarios. RCP 4.5 represents a moderate scenario with stabilization of radiative forcing by 2100^45^, while RCP 8.5 follows a “high-emission business as usual scenario”^46^. For this, additional climate data was acquired from ccafs-climate. org at a spatial resolution of 5 arcmin, covering the time steps of 2021–2040, 2041–2060 and 2061–2080. To account for uncertainties in climate modelling, data from 5 different global climate models (CESM 1 bcg, FIO ESM, GISS e2-r, INM CM4 and MPI-ESM-lr) were used for 5 separate sets of projections, from which a mean was then calculated for each time step and scenario. Mobility-Oriented Parity analysis (MOP) was applied in order to exclude potential bias in projections due to non-analogue climatic conditions^47^. Areas of low similarity to the calibration areas and strict extrapolation were consistently restricted to climatically extreme regions such as the Sahara and Atacama deserts as well as Greenland, where harsh climatic conditions would certainly exclude chikungunya anyway.

Human population density was deliberately not included as an explanatory variable for the climate-driven distribution model. Initial test runs showed that Chikungunya occurrence was (as expected) highly correlated with human population density, which dominated the models to a degree that climate effects were completely obfuscated. Instead, a post-hoc approach was applied that combines the results of the climate-driven models and human population density into a hazard index. For that, information on human population density was acquired from the Gridded Population of the World dataset^48^. On a 2.5 arcmin resolution raster, this dataset contains the predicted population density for the year 2015. To gain meaningful results, the population data was log-transformed. Afterwards values were scaled to a range between 0 and 1 to be comparable with the scale of the output of the climate-driven models. The 5 arcmin grid of the models was up-sampled to the finer 2.5 arcmin grid of the population data using a straight-forward “nearest neighbour” approach, and the two data sets were multiplied to gain a hazard index. For all future projections, human population density was held constant, as there were no reliable future projections of population development available for the whole study period and area.

## Electronic supplementary material

Supplementary Figures

Supplementary Data

## References

[1] Mavalankar D, Shastri P, Raman P (2007). Chikungunya epidemic in India: a major public-health disaster. Lancet Infect. Dis..

[2] Pialoux G, Gaüzère BA, Jauréguiberry S, Strobel M (2007). Chikungunya, an epidemic arbovirosis. Lancet Infect. Dis..

[3] Rezza G (2007). Infection with Chikungunya virus in Italy: an outbreak in a temperate region. Lancet.

[4] Grandadam M (2011). Chikungunya virus, southeastern France. Emerging Infect. Dis..

[5] Roiz, D., Boussès, P., Simard, F., Paupy, C. & Fontenille, D. Autochthonous Chikungunya transmission and extreme climate events in southern France. *PLoS Negl. Trop. Dis*. **9**, 10.1371/journal.pntd.0003854 (2015). 10.1371/journal.pntd.0003854 PMC4469319 26079620

[6] Wu D (2012). Chikungunya outbreak in Guangdong province, China, 2010. Emerging Infect. Dis..

[7] Van Bortel, W. *et al*. Chikungunya outbreak in the Caribbean region, December 2013 to March 2014, and the significance for Europe. *Euro. Surveill*. **19** (2014). 10.2807/1560-7917.es2014.19.13.20759 24721539

[8] Leparc-Goffart I, Nougairede A, Cassadou S, Prat C, De Lamballerie X (2014). Chikungunya in the Americas. Lancet.

[9] Morens DM, Fauci AS (2014). Chikungunya at the door - déjà vu all over again?. N. Engl. J. Med..

[10] Tsetsarkin, K. A. & Weaver, S. C. Sequential adaptive mutations enhance efficient vector switching by Chikungunya virus and its epidemic emergence. *PLoS Pathog*. **7**, doi:10.1371/journal.ppat.1002412 (2011). 10.1371/journal.ppat.1002412 PMC3234230 22174678

[11] Tatem AJ, Hay SI, Rogers DJ (2006). Global traffic and disease vector dispersal. Proc. Natl. Acad. Sci. USA..

[12] Semenza JC (2016). Determinants and drivers of infectious disease threat events in Europe. Emerging Infect. Dis..

[13] Tatem AJ (2012). Air travel and vector-borne disease movement. Parasitology.

[14] Campbell LP (2015). Climate change influences on global distributions of Dengue and Chikungunya virus vectors. Philos. Trans. R. Soc. B Biol. Sci..

[15] Brady, O. J. *et al*. Modelling adult Aedes aegypti and Aedes albopictus survival at different temperatures in laboratory and field settings. *Parasit. Vectors* **6**, doi:10.1186/1756-3305-6-351 (2013). 10.1186/1756-3305-6-351 PMC3867219 24330720

[16] Caminade C (2012). Suitability of European climate for the asian tiger mosquito Aedes albopictus: recent trends and future scenarios. J. R. Soc. Interface.

[17] Fischer D, Thomas SM, Niemitz F, Reineking B, Beierkuhnlein C (2011). Projection of climatic suitability for Aedes albopictus Skuse (Culicidae) in Europe under climate change conditions. Glob. Planet. Change.

[18] Fischer, D., Thomas, S. M., Neteler, M., Tjaden, N. B. & Beierkuhnlein, C. Climatic suitability of Aedes albopictus in Europe referring to climate change projections: comparison of mechanistic and correlative niche modelling approaches. *Euro. Surveill*. **19** (2014). 10.2807/1560-7917.es2014.19.6.20696 24556349

[19] Fischer, D. *et al*. Climate change effects on chikungunya transmission in europe: geospatial analysis of vector’s climatic suitability and virus’ temperature requirements. *Int. J. Health Geogr*. **12**, doi:10.1186/1476-072X-12-51 (2013). 10.1186/1476-072X-12-51 PMC3834102 24219507

[20] Ruiz-Moreno D, Vargas IS, Olson KE, Harrington LC (2012). Modeling dynamic introduction of Chikungunya virus in the United States. PLoS Negl. Trop. Dis..

[21] Bhatt S (2013). The global distribution and burden of Dengue. Nature.

[22] Nsoesie EO (2016). Global distribution and environmental suitability for Chikungunya virus, 1952 to 2015. Euro. Surveill..

[23] Samy AM, Thomas SM, Wahed AA, Cohoon KP, Peterson AT (2016). Mapping the global geographic potential of Zika virus spread. Mem. Inst. Oswaldo Cruz.

[24] Samy, A. M., van de Sande, W. W. J., Fahal, A. H. & Peterson, A. T. Mapping the potential risk of Mycetoma infection in Sudan and South Sudan using ecological niche modeling. *PLoS Negl. Trop. Dis*. **8** (2014). 10.1371/journal.pntd.0003250 PMC4199553 25330098

[25] Moo Llanes DA (2016). Nicho ecológico actual y futuro de la Leishmaniasis (Kinetoplastida: Trypanosomatidae) en la región Neotropical. Rev. Biol. Trop..

[26] Peterson AT, Samy AM (2016). Geographic potential of disease caused by Ebola and Marburg viruses in Africa. Acta Trop..

[27] van Vuuren D (2011). The representative concentration pathways: an overview. Clim. Change.

[28] Hijmans RJ, Cameron SE, Parra JL, Jones PG, Jarvis A (2005). Very high resolution interpolated climate surfaces for global land areas. Int. J. Climatol..

[29] Semenza JC, Menne B (2009). Climate change and infectious diseases in Europe. Lancet Infect. Dis..

[30] Parham PE (2015). Climate, environmental and socio-economic change: weighing up the balance in vector-borne disease transmission. Philos. Trans. R. Soc. B Biol. Sci..

[31] Lafferty KD (2009). The ecology of climate change and infectious diseases. Ecology.

[32] Suk JE (2016). Climate change, malaria, and public health: accounting for socioeconomic contexts in past debates and future research. WIREs Clim. Change.

[33] Rogers DJ, Randolph SE (2000). The global spread of Malaria in a future, warmer world. Science.

[34] Halstead SB (2015). Reappearance of Chikungunya, formerly called Dengue, in the Americas. Emerging Infect. Dis..

[35] Mordecai, E. *et al*. Detecting the impact of temperature on transmission of Zika, Dengue and Chikungunya using mechanistic models. *PLos Negl. Trop. Dis.* **11**, e000556, doi:10.1371/journal.pntd.0005568 (2017). 10.1371/journal.pntd.0005568 PMC5423694 28448507

[36] Kraemer MUG (2015). The global distribution of the arbovirus vectors Aedes aegypti and Ae. albopictus. Elife.

[37] Thomas, S. M., Obermayr, U., Fischer, D., Kreyling, J. & Beierkuhnlein, C. Low-temperature threshold for egg survival of a post-diapause and non-diapause European aedine strain, Aedes albopictus (Diptera: Culicidae). *Parasit. Vectors* **5** (2012). 10.1186/1756-3305-5-100 PMC3403971 22621367

[38] Tjaden, N. B., Thomas, S. M., Fischer, D. & Beierkuhnlein, C. Extrinsic incubation period of Dengue: knowledge, backlog, and applications of temperature dependence. *PLoS Negl. Trop. Dis* **7** (2013). 10.1371/journal.pntd.0002207 PMC3694834 23826399

[39] Phillips SJ, Anderson RP, Schapire RE (2006). Maximum entropy modeling of species geographic distributions. Ecol. Model..

[40] VanDerWal J, Shoo LP, Graham C, William SE (2009). Selecting pseudo-absence data for presence-only distribution modeling: How far should you stray from what you know?. Ecol. Model..

[41] Barve N (2011). The crucial role of the accessible area in ecological niche modeling and species distribution modeling. Ecol. Model..

[42] Elith J, Kearney M, Phillips S (2010). The art of modelling range-shifting species. Methods Ecol. Evol..

[43] Elith J (2011). A statistical explanation of MaxEnt for ecologists. Divers. Distrib..

[44] Peterson AT, Papes M, Soberon J (2008). Rethinking receiver operating characteristic analysis applications in ecological niche modeling. Ecol. Model..

[45] Thomson AM (2011). RCP4.5: a pathway for stabilization of radiative forcing by 2100. Clim. Change..

[46] Riahi K (2011). RCP 8.5-A scenario of comparatively high greenhouse gas emissions. Clim. Change..

[47] Owens, H. *et al*. Constraints on interpretation of ecological niche models by limited environmental ranges on calibration areas. *Ecol. Model.* **263**, 10–18 (2013).

[48] Center for International Earth Science Information Network - CIESIN - Columbia University & Centro Internacional de Agricultura Tropical - CIAT. Gridded population of the world, version 3 (GPWv3): population density grid, future estimates. *Palisades, NY: NASA Socioeconomic Data and Applications Center* (*SEDAC*), doi:10.7927/H4ST7MRB (2005).

