# Supplementary material for: Modelling the effects of global climate change on Chikungunya transmission in the 21st century
Source: Sci Rep. 2017 Jun 19;7:3813. doi: 10.1038/s41598-017-03566-3 (PMC5476675; doi:10.1038/s41598-017-03566-3)
Supplement: Supplementary file 1 — Supplementary Figures [file 41598_2017_3566_MOESM1_ESM.pdf]

Modelling global climate change effects on Chikungunya in the 21st century

Nils Tjaden, Jonathan Suk, Dominik Fischer, Stephanie Thomas, Carl Beierkuhnlein, Jan Semenza

Supplementary figures

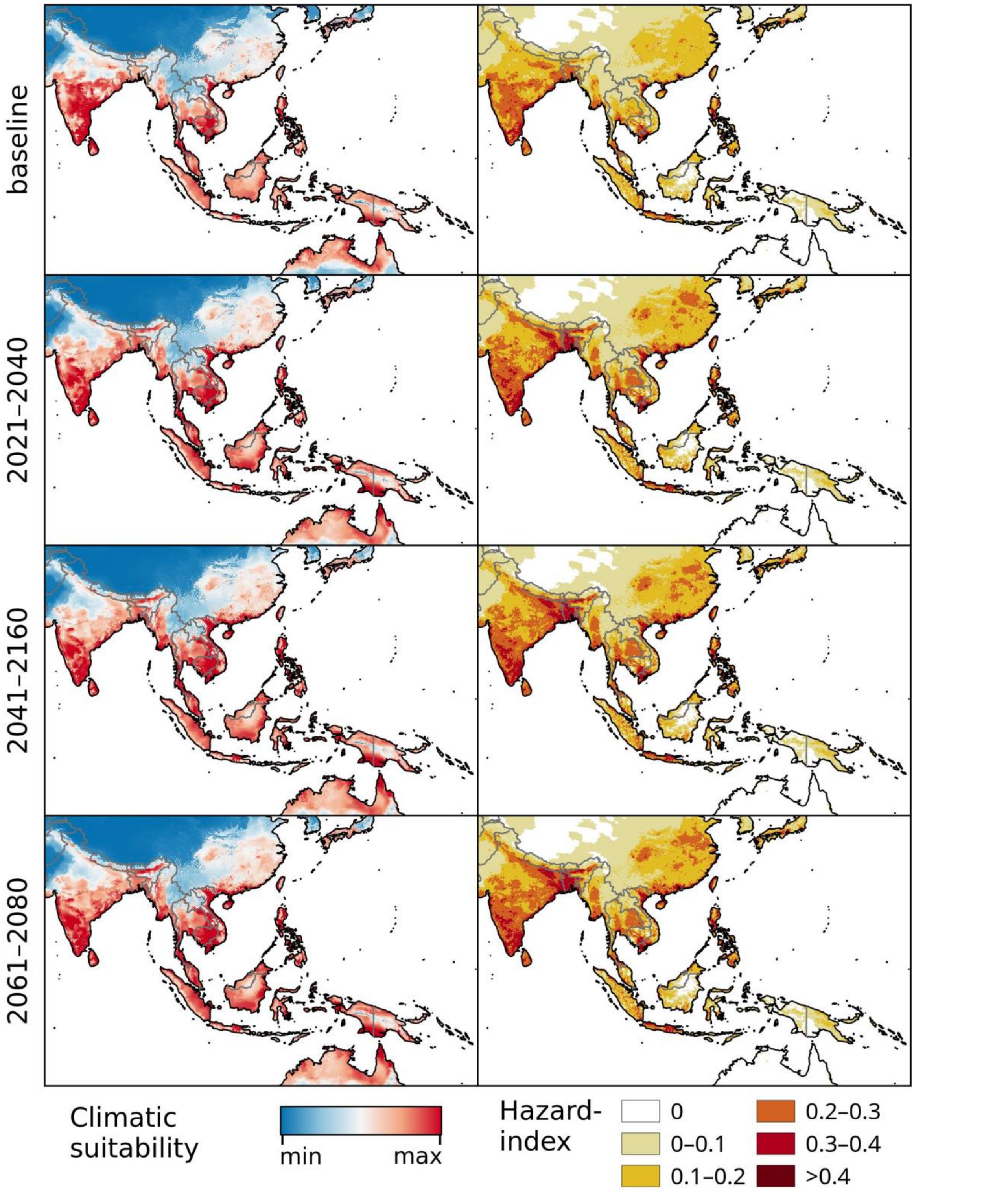

Supplementary Figure S1: Chikungunya under the baseline and RCP 4.5 climate change scenarios in Asia and Australasia. Left: Climatic suitability, right: hazard index. Climate change scenarios represent the mean model output obtained through the 5 GCMs. Climatic suitability output is scaled to the over-all global minimum (0) and maximum (0.623) values observed in any model. Maps were generated using the “raster” package in R 3.3.2 (https://www.r-project.org/) and QGIS 2.8.1 (https://www.qgis.org/).

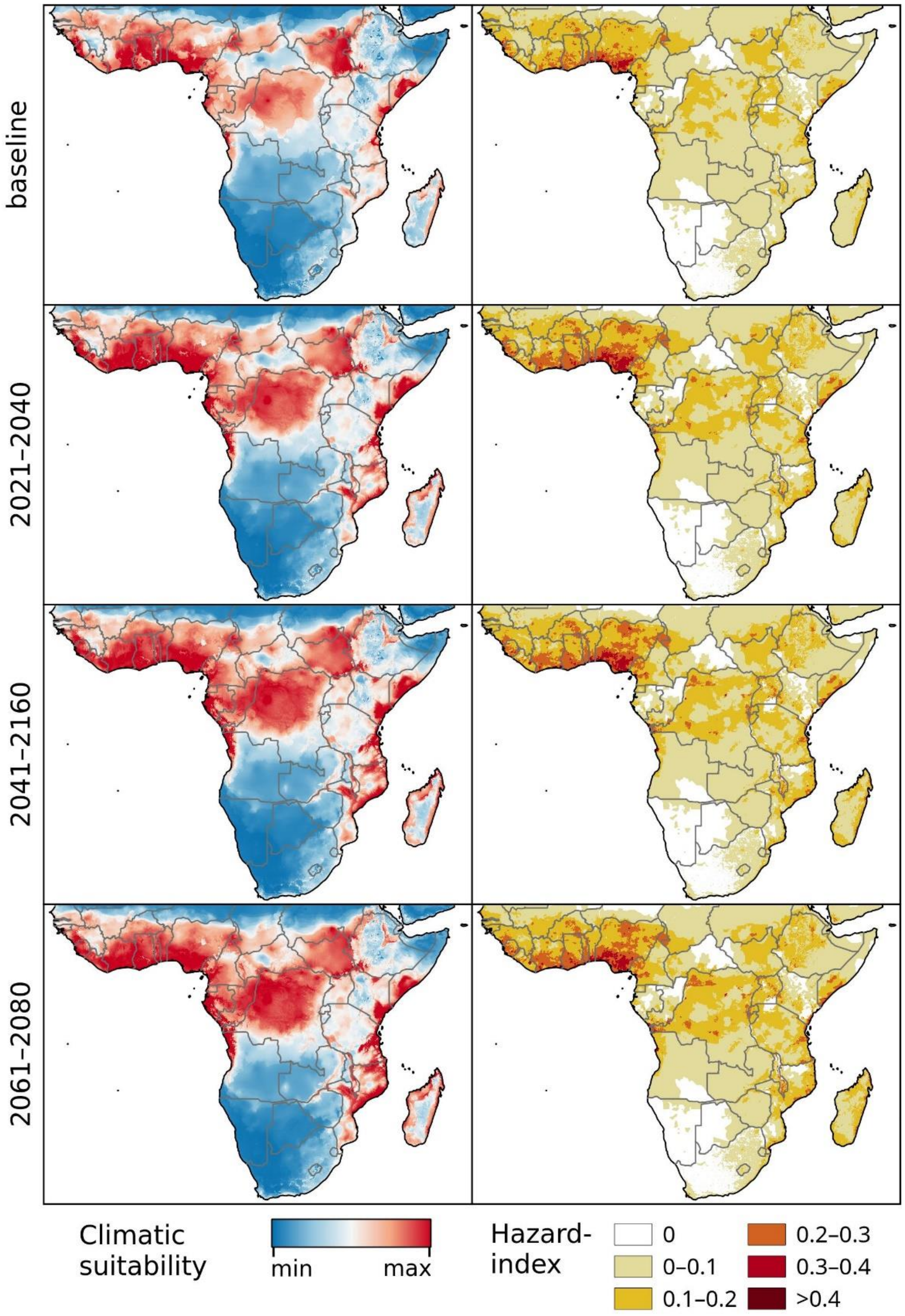

Supplementary Figure S2: Chikungunya under the baseline and RCP 4.5 climate change scenarios in Africa. Left: Climatic suitability, right: hazard index. Climate change scenarios represent the mean model output obtained through the 5 GCMs. Climatic suitability output is scaled to the over-all global minimum (0) and maximum (0.623) values observed in any model. Maps were generated using the "raster" package in R 3.3.2 (https://www.r-project.org/) and QGIS 2.8.1 (https://www.qgis.org/).

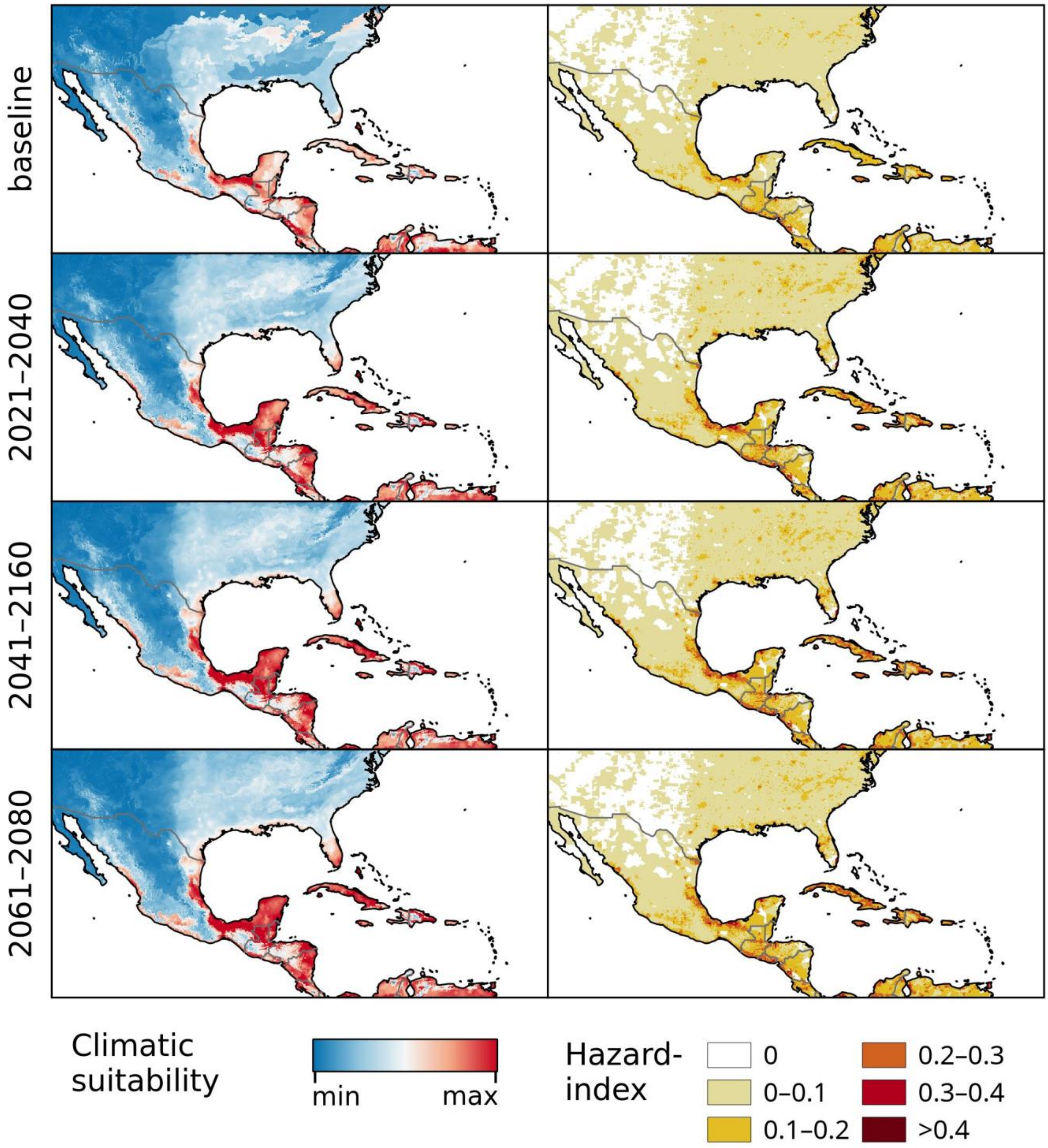

Supplementary Figure S3: Chikungunya under the baseline and RCP 4.5 climate change scenarios in North- and Central America. Left: Climatic suitability, right: hazard index. Climate change scenarios represent the mean model output obtained through the 5 GCMs. Climatic suitability output is scaled to the over-all global minimum (0) and maximum (0.623) values observed in any model. Maps were generated using the "raster" package in R 3.3.2 (https://www.r-project.org/) and QGIS 2.8.1 (https://www.qgis.org/).

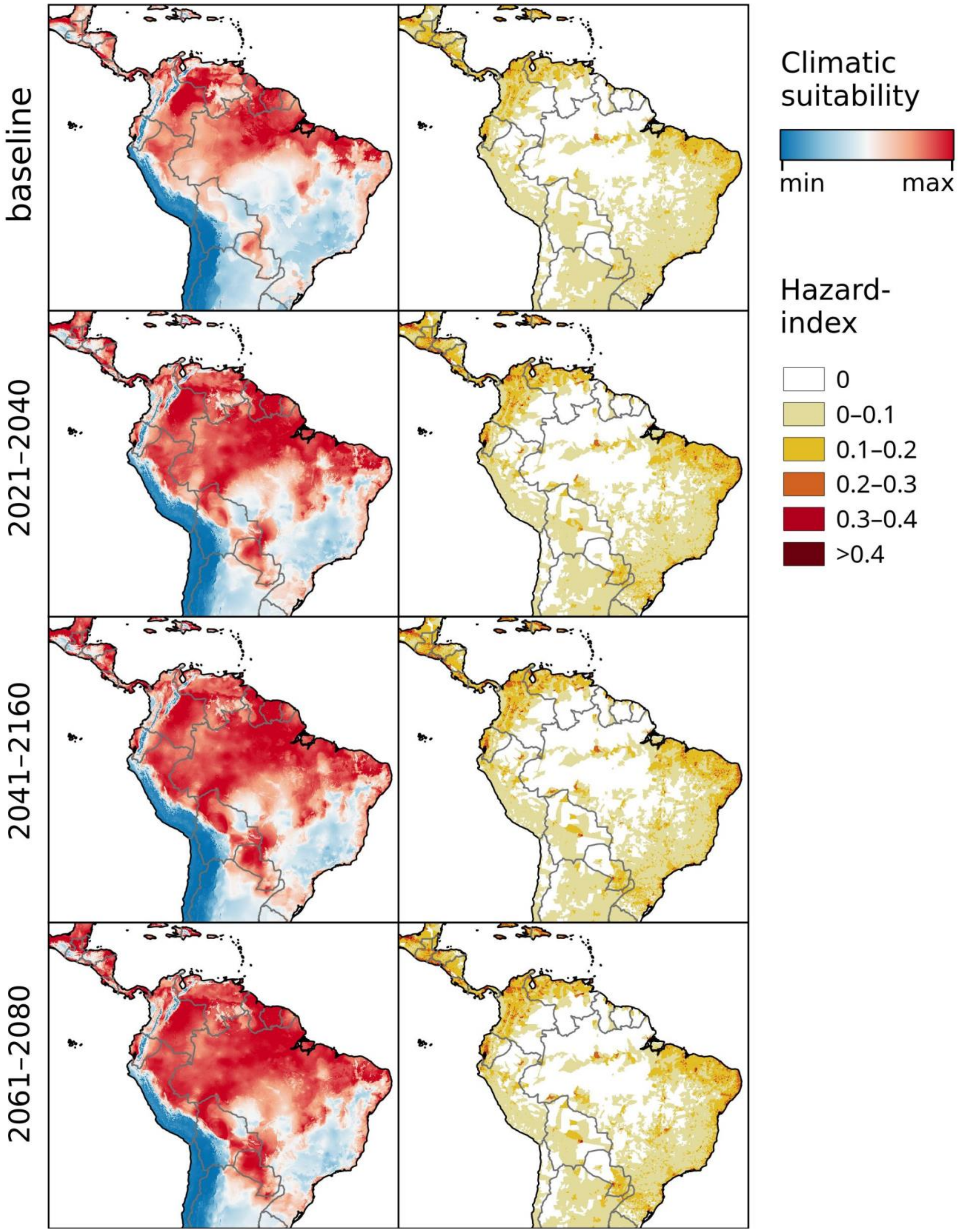

Supplementary Figure S4: Chikungunya under the baseline and RCP 4.5 climate change scenarios in South America. Left: Climatic suitability, right: hazard index. Climate change scenarios represent the mean model output obtained through the 5 GCMs. Climatic suitability output is scaled to the over-all global minimum (0) and maximum (0.623) values observed in any model. Maps were generated using the "raster" package in R 3.3.2 (https://www.r-project.org/) and QGIS 2.8.1 (https://www.qgis.org/).

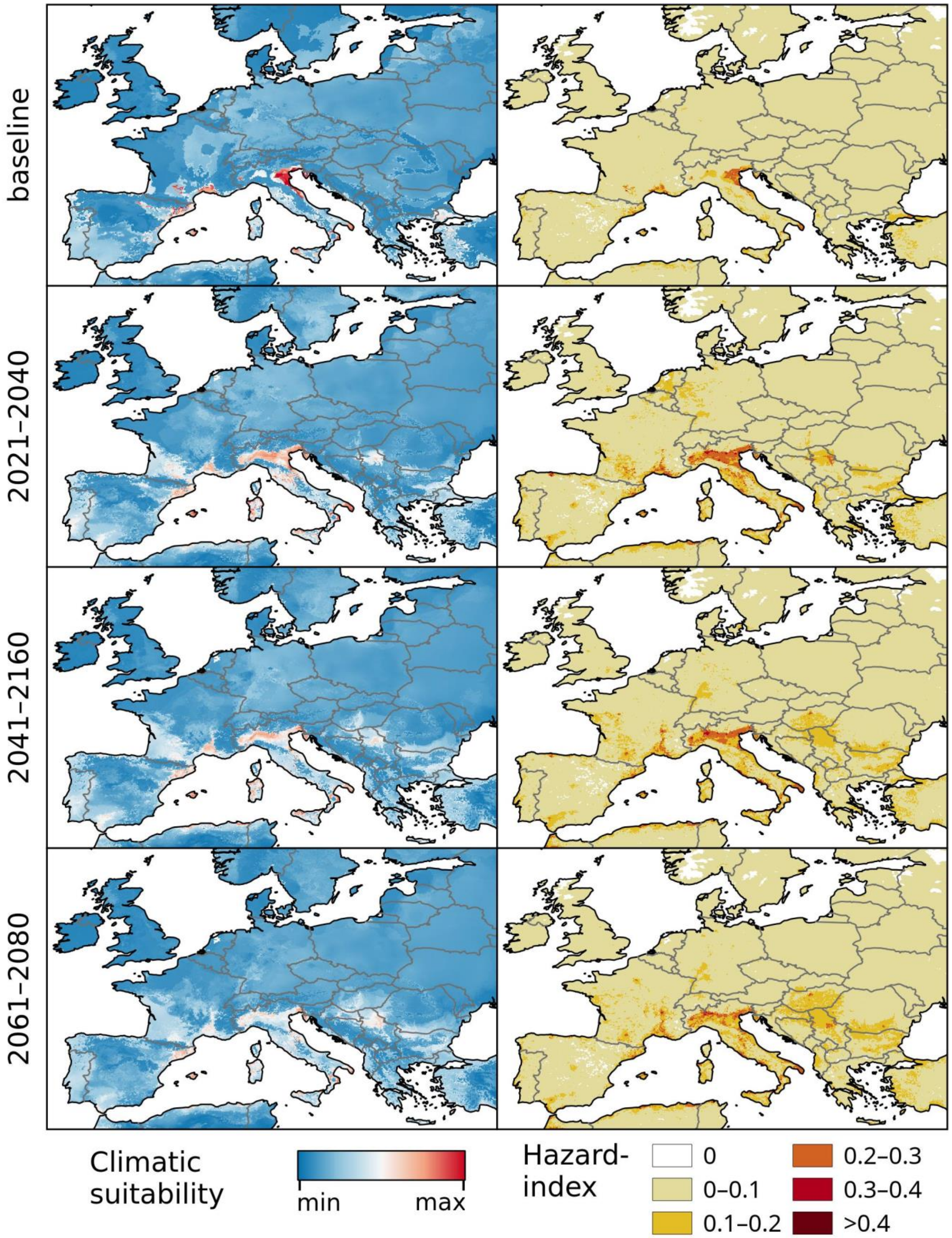

Supplementary Figure S5: Chikungunya under the baseline and RCP 4.5 climate change scenarios in Europe. Left: Climatic suitability, right: hazard index. Climate change scenarios represent the mean model output obtained through the 5 GCMs. Climatic suitability output is scaled to the over-all global minimum (0) and maximum (0.623) values observed in any model. Maps were generated using the “raster” package in R 3.3.2 (https://www.r-project.org/) and QGIS 2.8.1 (https://www.qgis.org/).

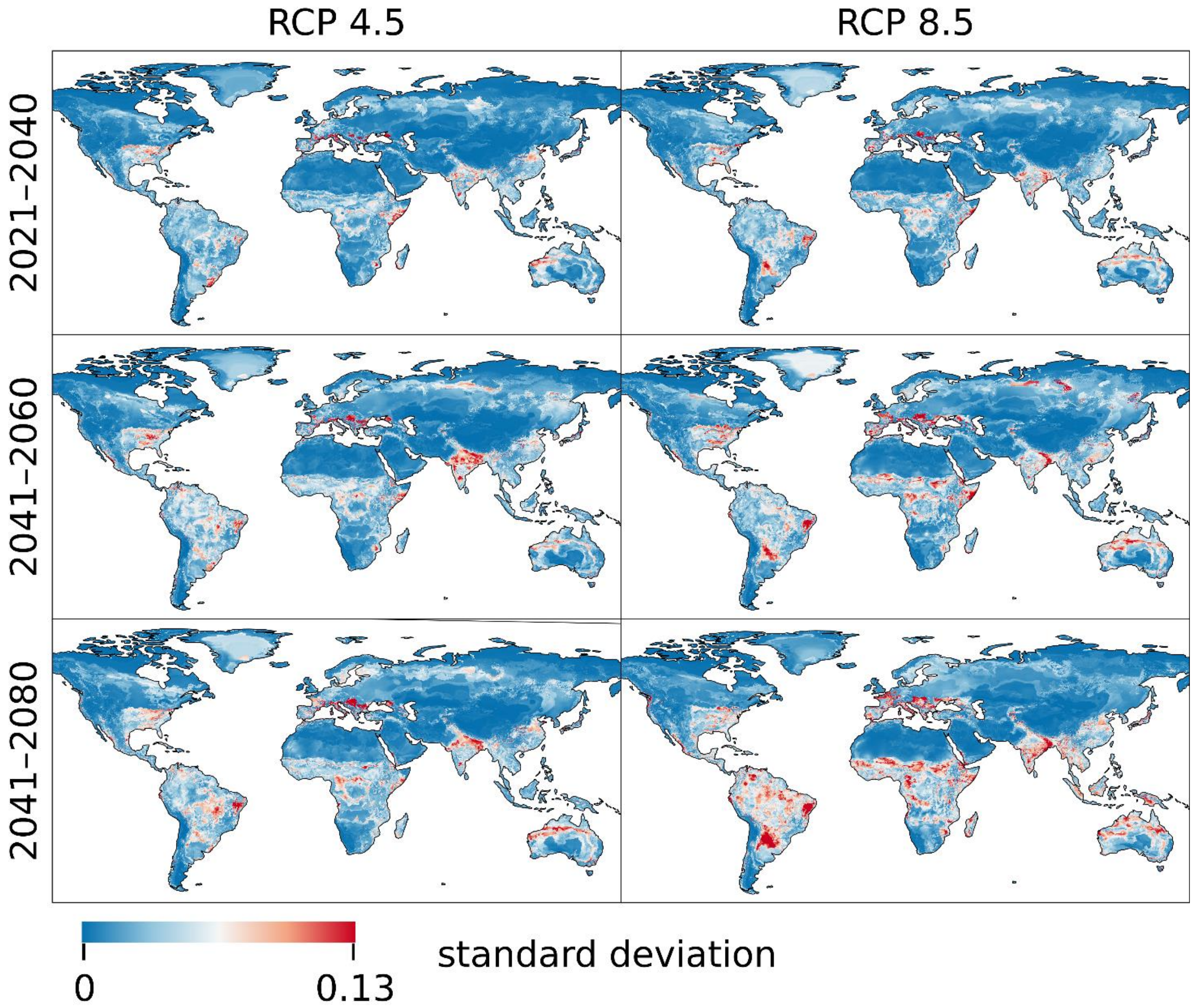

Supplementary Figure S6: Standard deviation of future projections across 5 global climate models. Maps were generated using the “raster” package in R 3.3.2 (https://www.r-project.org/) and QGIS 2.8.1 (https://www.qgis.org/).

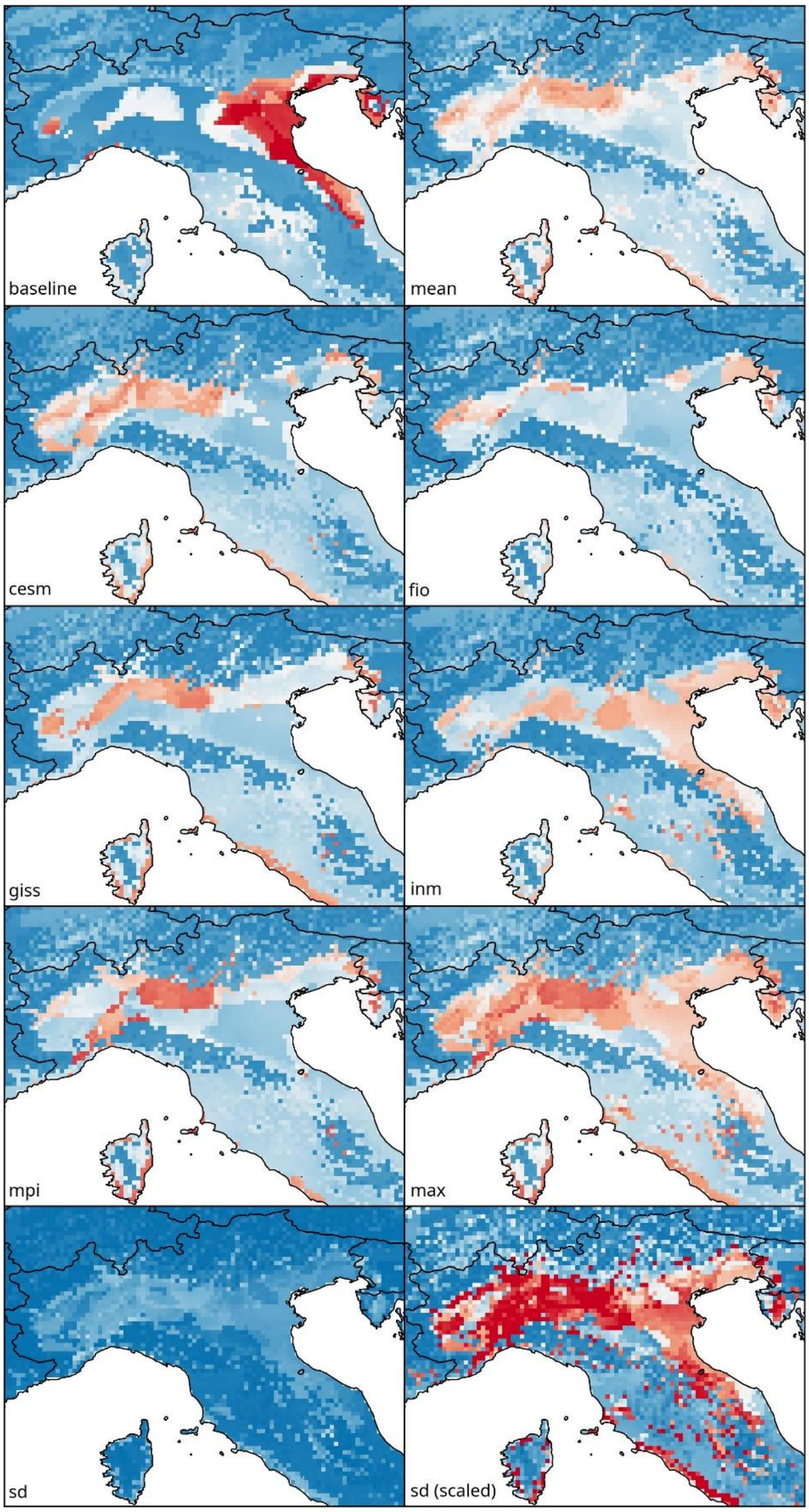

This figure shows how at high zoom factors small-scale differences in how different global climate models (GCM) project future climate conditions can affect the projection of future climatic suitability when viewed as a mean value of several models.
For the Po valley in northern Italy shown here, projections from all 5 GCM show suitable areas in the valley. The exact location of those suitable areas within the valley, however, varies from GCM to GCM. When these areas are summarized in a mean value, the suitable area appears to shrink compared to the baseline model. In this case, the using the maximum value of all 5 GCMs instead is likely to paint a more realistic picture.

Apart from "baseline", all data shown is for RCP 8.5 and the 2021–2040 timestep. From left to right, top to bottom:

baseline = original model based on worldclim
mean = mean suitability over all 5 GCM

cesm = cesm1 bcg
fio = fio esm
giss = giss e2 r
inm = inm cm4
mpi = mpi esm lr

max = maximum suitability of over 5 GCM
sd = standard deviation of all 5 GCM at the same scale as model output
sd (scaled) = same data as "sd", but scaled so that maximum red refers to maximum sd observed in this section of the global model

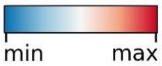

Supplementary Figure S7: Comparison of small-scale variations in projected future climatic suitability of Chikungunya based on different climate models. Small scale differences in projected climate may lead to local under-estimations of climatic suitability. This is especially apparent for the Po Valley in northern Italy: all projections obtained from the 5 GCMs agree that there are highly suitable areas in this region, but the location of those areas within the region varies between GCMs, leading to a lower than expected mean suitability. Maps were generated using the “raster” package in R 3.3.2 (https://www.r-project.org/) and QGIS 2.8.1 (https://www.qgis.org/).

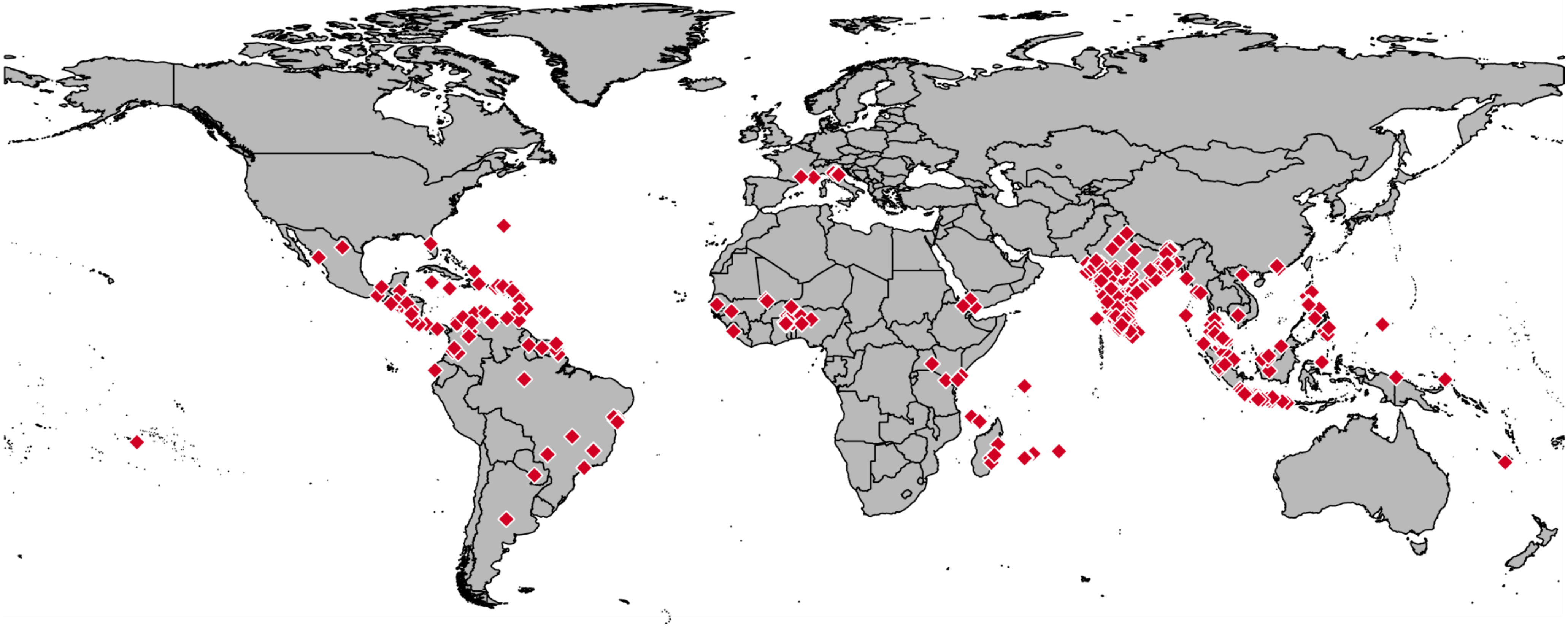

Supplementary Figure S8: Global map of Chikungunya occurrences used to train the models. The map was generated using QGIS 2.8.1 (https://www.qgis.org/).
